# Evaluation of anti-PD-1-based therapy against triple-negative breast cancer patient-derived xenograft tumors engrafted in humanized mouse models

**DOI:** 10.1186/s13058-018-1037-4

**Published:** 2018-09-05

**Authors:** Roberto R. Rosato, Daniel Dávila-González, Dong Soon Choi, Wei Qian, Wen Chen, Anthony J. Kozielski, Helen Wong, Bhuvanesh Dave, Jenny C. Chang

**Affiliations:** Houston Methodist Cancer Center, Houston Methodist Hospital, Houston, TX 77030 USA

**Keywords:** Triple-negative breast cancer, TNBC, Immunotherapy, Anti-PD-1, PD-L1, Humanized mouse model

## Abstract

**Background:**

Breast cancer has been considered not highly immunogenic, and few patients benefit from current immunotherapies. However, new strategies are aimed at changing this paradigm. In the present study, we examined the in vivo activity of a humanized anti-programmed cell death protein 1 (anti-PD-1) antibody against triple-negative breast cancer (TNBC) patient-derived xenograft (PDX) tumor models.

**Methods:**

To circumvent some of the limitations posed by the lack of appropriate animal models in preclinical studies of immunotherapies, partially human leukocyte antigen-matched TNBC PDX tumor lines from our collection, as well as human melanoma cell lines, were engrafted in humanized nonobese diabetic/severe combined immunodeficiency *IL2R*γ^*null*^ (hNSG) mice obtained by intravenous injection of CD34^+^ hematopoietic stem cells into nonlethally irradiated 3–4-week-old mice. After both PDXs and melanoma cell xenografts reached ~ 150–200 mm^3^, animals were treated with humanized anti-PD-1 antibody or anti-CTLA-4 and evaluated for tumor growth, survival, and potential mechanism of action.

**Results:**

Human CD45^+^, CD20^+^, CD3^+^, CD8^+^, CD56^+^, CD68^+^, and CD33^+^ cells were readily identified in blood, spleen, and bone marrow collected from hNSG, as well as human cytokines in blood and engrafted tumors. Engraftment of TNBC PDXs in hNSG was high (~ 85%), although they grew at a slightly slower pace and conserved their ability to generate lung metastasis. Human CD45^+^ cells were detectable in hNSG-harbored PDXs, and consistent with clinical observations, anti-PD-1 antibody therapy resulted in both a significant reduction in tumor growth and increased survival in some of the hNSG PDX tumor lines, whereas no such effects were observed in the corresponding non-hNSG models.

**Conclusions:**

This study provides evidence associated with anti-PD-1 immunotherapy against TNBC tumors supporting the use of TNBC PDXs in humanized mice as a model to overcome some of the technical difficulties associated with the preclinical investigation of immune-based therapies.

**Electronic supplementary material:**

The online version of this article (10.1186/s13058-018-1037-4) contains supplementary material, which is available to authorized users.

## Background

Immunotherapy has revolutionized the treatment regimens for various cancer types, leading to improved clinical responses in otherwise untreatable advanced cancers [1]. Observations showing accumulation of tumor-infiltrating lymphocytes (TILs) within the tumor microenvironment (TME), as well as work highlighting the efficacy of immune checkpoint inhibitors (CPIs), have sparked interest in the further development of these approaches. Studies have focused on the development of CPIs, including cytotoxic T-lymphocyte-associated protein 4 (CTLA-4) [2, 3] as well as programmed cell death 1 (PD-1) receptor and its ligands programmed death ligand 1 (PD-L1) and PD-L2 [4–6]. PD-1 is found on cytotoxic T cells and T-regulatory cells and is expressed when T cells become activated in response to inflammation or infection in peripheral tissues [7, 8]. Binding of the PD-1 ligand to its receptor inactivates the T cell, limiting the immune response to the stimuli, thereby causing immune suppression [7, 8]. Cancer cells, however, induce PD-1 L expression, enhancing the immunosuppressive action of this pathway, ultimately allowing them to “hide” from natural immune attack [7, 8]. Anti-PD-1/PD-L1 therapies disrupt this pathway by preventing these interactions, leaving activated cytotoxic T cells available to attack the cancer cells [7, 8]. In triple-negative breast cancer (TNBC), a minority of patients benefit from these approaches, and further studies are urgently needed, especially those designed to evaluate combinatorial therapies.

The recent evolution of these therapeutic strategies (i.e., allowing the immune system to identify neoplastic growth in order to prevent carcinogenesis and eliminate cancer cells) has led to the urgent need for having available a range of appropriate small-animal models that may serve in testing these interactions [9, 10]. To this end, mouse models injected with human CD34^+^ hematopoietic stem cells (HSCs; “humanized” mice) are currently commercially available for studies in cancer, infectious diseases, and gene therapy, among others. However, these models remain relatively expensive, beyond the means of most academic laboratories, especially when used in large-scale studies.

Important advances have been made in the recent years in establishing mouse models to be used in cancer-related studies, including patient-derived xenografts (PDXs). PDXs, by conserving the characteristic of the human primary tumor, are useful for addressing critical questions regarding tumor biology and response to newly developed therapeutic concepts [11, 12]. In contrast to cell lines used for in vivo studies, PDXs retain morphology, cellular heterogeneity, and molecular profiles of the original patient tumors [12–18], representing an effective model for screening potential chemotherapeutics and translating them to enhanced efficacy in clinical trials [19–22]. New experimental designs have recently been used as valid approaches to perform large-scale PDX-based preclinical trials to evaluate and predict the clinical efficacy and drug response of new therapeutics following the so-called 1 × 1 × 1 design [15, 23, 24]. By using this design (i.e., one animal per model per treatment), PDX models provide the ability to place the same “patient” on all arms of a trial in a given preclinical study.

We have developed an extensive cohort of breast cancer PDXs that retain the morphology, cellular heterogeneity, and molecular profiles of the original patient tumors, serving as a renewable, quality-controlled tissue resource for preclinical evaluation of novel treatment regimens for what are in some cases extremely aggressive cancer types that currently lack adequate targeted therapeutic options [12]. These PDXs have been characterized and classified according to Perou PAM50 and Pietenpol subtypes [11, 25, 26] and their *TP53* mutational status [11, 12, 27]. However, new therapies involving, among others, immune CPIs emphasize the need for the appropriate small-animal models to examine xenograft growth and response to therapy in the context of a “human” immune system and TME.

In the present study, we investigated the in vivo activity of anti-immune CPI-based therapies against TNBC PDX tumor models established in models of “humanized” nonobese diabetic/severe combined immunodeficiency *IL2R*γ^*null*^ (hNSG) mice by the engraftment of human CD34^+^ HSCs, as previously described [28, 29]. We show that, in terms of the animal model, engrafted human HSCs displayed self-renewal and multilineage differentiation capacities and that anti-PD-1 antibody therapy may result, as observed in clinical studies, in varying effects, with some PDXs responding positively to the treatment (i.e., significant reduction in tumor growth and increased survival), whereas others show no signs of improvement. Importantly, in those models that responded to the anti-PD-1 therapy, the effects were differentially displayed and observed only in the hNSG mice, indicating that despite potential limitations of the model, it may still represent an important tool for the preclinical evaluation of immunotherapies in breast cancer.

## Methods

### Mice

All the present study protocols involving mice followed the standard regulations and were approved by the Houston Methodist Research Institute Institutional Animal Care and Use Committee. “Humanized” mouse models refer to immunodeficient mice engrafted with human hematopoietic and lymphoid cells or tissues. NOD.Cg-*Prkdc*^*scid*^ *Il2rg*^*tm1Wjl*^/SzJ (NOD scid γ [NSG]; The Jackson Laboratory, Bar Harbor, ME, USA) mice were used as the recipient strain to intravenously (i.v.) engraft human CD34^+^ HSCs (STEMCELL Technologies, Vancouver, BC, Canada) as previously described [28, 29]. Briefly, 21-day-old NSG mice were irradiated with 240 cGy (sublethal) whole-body γ-irradiation. After 4–6 hours, mice were inoculated via the lateral tail vein with 3 × 10^4^ CD34^+^ HSCs. HSCs were allowed to engraft, and peripheral blood of recipient mice was collected from the retro-orbital sinus and analyzed by flow cytometry as indicated in the corresponding figure legends herein. “hNSG” is used to denote that the mice have HSC cells engrafted.

PDXs were originally derived by transplanting a fresh patient breast tumor biopsy into the cleared mammary gland fat pad of immunocompromised mice. Tumor samples (2 × 2 mm) were serially passaged in NSG mice by fat pad transplant under general anesthesia [12]. Low-passage TNBC MC1 [30], BCM-2147, BCM-4913, BCM-4664, and BCM-5471 [12] samples were transferred into hNSG mice for engraftment approximately 6–8 weeks after initial human CD34^+^ HSC cells tail vein injection. The weight of the mice was recorded and tumor volumes were measured and calculated [0.5 × (long dimension) × (short dimension)^2^] twice weekly. When tumors reached an average size of 150–200 mm^3^, mice were randomized (*n* ≥ 5 per group) and used to determine the response to the treatment.

As validation of the humanized model, immunogenic A375 melanoma cell lines (American Type Culture Collection, Manassas, VA, USA) were maintained in DMEM (Life Technologies, Carlsbad, CA, USA), 10% FBS (HyClone; Life Technologies), and 1% antibiotic-antimycotic in a humidified 5% CO_2_ incubator at 37 °C. Cells (5 × 10^5^) were injected orthotopically into the skin of NSG and hNSG mice and after 7–10 days (palpable tumors), and mice were randomly sorted into treatment groups.

### Reagents

Humanized antibodies were obtained from Merck Oncology (Kenilworth, NJ, USA; pembrolizumab [Keytruda™], anti-PD-1) and Bristol-Myers Squibb (New York, NY, USA; nivolumab [Opdivo™], anti-PD-1; and ipilimumab, anti-CTL-4). Serum and tumor contents of human cytokine and chemokine biomarkers were determined by using the MILLIPLEX MAP Human High Sensitivity T Cell Panel Premixed 13-plex, Immunology Multiplex Assay (EMD Millipore, Billerica, MA, USA). Lymphoprep (STEMCELL Technologies) was used to isolate human peripheral blood mononuclear cells from tumor.

### IHC

IHC assays were performed following established protocols [31]. After antigen retrieval (Tris-Cl, pH 9.0), paraffin-embedded sections of PDX tumors were incubated for 1 hour at room temperature with the following antibodies: antihuman CD45 (leukocyte common antigen, clones 2B11 + PD7/26); antihuman CD68, clone KP1; antihuman CD8 (clone C8/144B); antihuman CD4, clone 4B12; antihuman Ki-67, clone MIB-1 (Dako, Glostrup, Denmark); antihuman CD3, clone UCHT1 (STEMCELL Technologies); antihuman CD20, clone EP459Y; antihuman CD56, clone EPR2566 (Abcam, Cambridge, MA, USA); antihuman cytokeratin 19 (CK19), clone A53-B/A2.26, also known as Ks19.1 (Thermo Scientific, Waltham, MA, USA).

### Western blot analysis

Protein analysis was performed by Western blotting [31]. Briefly, whole-cell lysates were made in 1× lysis buffer (Cell Signaling Technology, Danvers, MA, USA) with protease/phosphatase inhibitor cocktail (Thermo Scientific). Samples (30 μg) were boiled in sample buffer (Thermo Scientific) containing β-mercaptoethanol (Sigma-Aldrich, St. Louis, MO, USA) and subjected to SDS-PAGE electrophoresis in 4–20% polyacrylamide gels (Bio-Rad Laboratories, Hercules, CA, USA), transferred onto nitrocellulose membranes (Bio-Rad Laboratories), and incubated overnight at 4 °C with primary antibodies (1:1000; anti-PD-L1, catalogue no. 13684; anti-β-actin, catalogue no. 4970; Cell Signaling Technology), followed after washes by the appropriate secondary antibodies for 1 hour (1:2000). Protein bands were developed in autoradiography films (Denville Scientific Inc., South Plainfield, NJ, USA).

### Fluorescence-activated cell sorting analysis

Analysis of mouse and human blood, spleen, and bone marrow mononuclear cells was performed by fluorescence-activated cell sorting analysis [29, 32]. The antibodies used were as follows: antimouse CD45-fluorescein isothiocyanate (FITC), clone 30-F11; antihuman CD45-allophycocyanin (APC), clone HI30; antihuman CD3-phycoerythrin (PE), clone UCHT1; antihuman CD20-FITC, clone 2H7; PE-cyanine 7 mouse antihuman CD68, clone Y1/82A; Alexa Fluor 700 mouse antihuman CD56, clone B159; antimouse CD45-PE, clone 30-F11; antimouse CD45-peridinin chlorophyll protein complex, clone 30-F11; mouse immunoglobulin G2b (IgG2b), κ isotype-FITC, clones 27–35; mouse IgG1, κ isotype-PE, clone MOPC-21; and mouse IgG2b κ isotype-APC (BD Biosciences, San Jose, CA, USA); Pacific Blue antihuman CD33 eFluor® 450, clone P67; and Pacific Blue Mouse IgG1 K Isotype Control eFluor® 450 (eBioscience, San Diego, CA, USA). Briefly, erythrocytes were lysed, after which lymphoid cells were incubated with the corresponding antibodies and fixed following standard procedures [29, 32]. Flow cytometric analysis was performed at the Houston Methodist Research Institute Flow Cytometry Core using a BD LSRFortessa flow cytometer for acquisition of data and FACSDiva software (both from BD Biosciences) for analysis.

### Tumor-infiltrating lymphocyte cytotoxic activity assay

Following a four-cycle treatment with anti-PD-1 antibody (nivoluzumab 10 mg/kg), MC1-engrafted tumors growing in hNSG mice were collected and mechanically disaggregated into single cells, and TILs were isolated by using Ficoll gradient (Lymphoprep; STEMCELL Technologies). These TILs were cocultured with MC1 tumor cells extracted from nonhumanized NSG mice for 6 hours (250:7 ratio of target cells to effector cells), and TIL cytotoxic activity was measured with the CytoTox 96® Non-Radioactive Cytotoxicity Assay (Promega, Madison, WI, USA) as per the manufacturer’s instructions. Granzyme B tumor levels were measured by incubating tumor protein lysates with antibody-immobilized magnetic beads (HGRNZMB-MAG; EMD Millipore, Billerica, MA) and evaluated using a Luminex LX-200 multiplexing assay system (Luminex Corp., Austin, TX, USA).

### Statistical analysis

All data were analyzed using Prism software (GraphPad Software, La Jolla, CA, USA). Data are presented as mean ± SEM. Statistical significance between two groups was analyzed by two-tailed Student’s *t* test. Experiments with more than three groups were analyzed with one-way analysis of variance (ANOVA) and Bonferroni’s post hoc test. Statistical analysis of tumor volume was assessed by two-way ANOVA and Bonferroni’s post hoc test. Survival proportions were assessed by using the Kaplan-Meier method and further analyzed with either Wilcoxon or log-rank test. A *P* value less than 0.05 was considered significant.

## Results

### Establishment of hNSG models

**Fig. 1:**
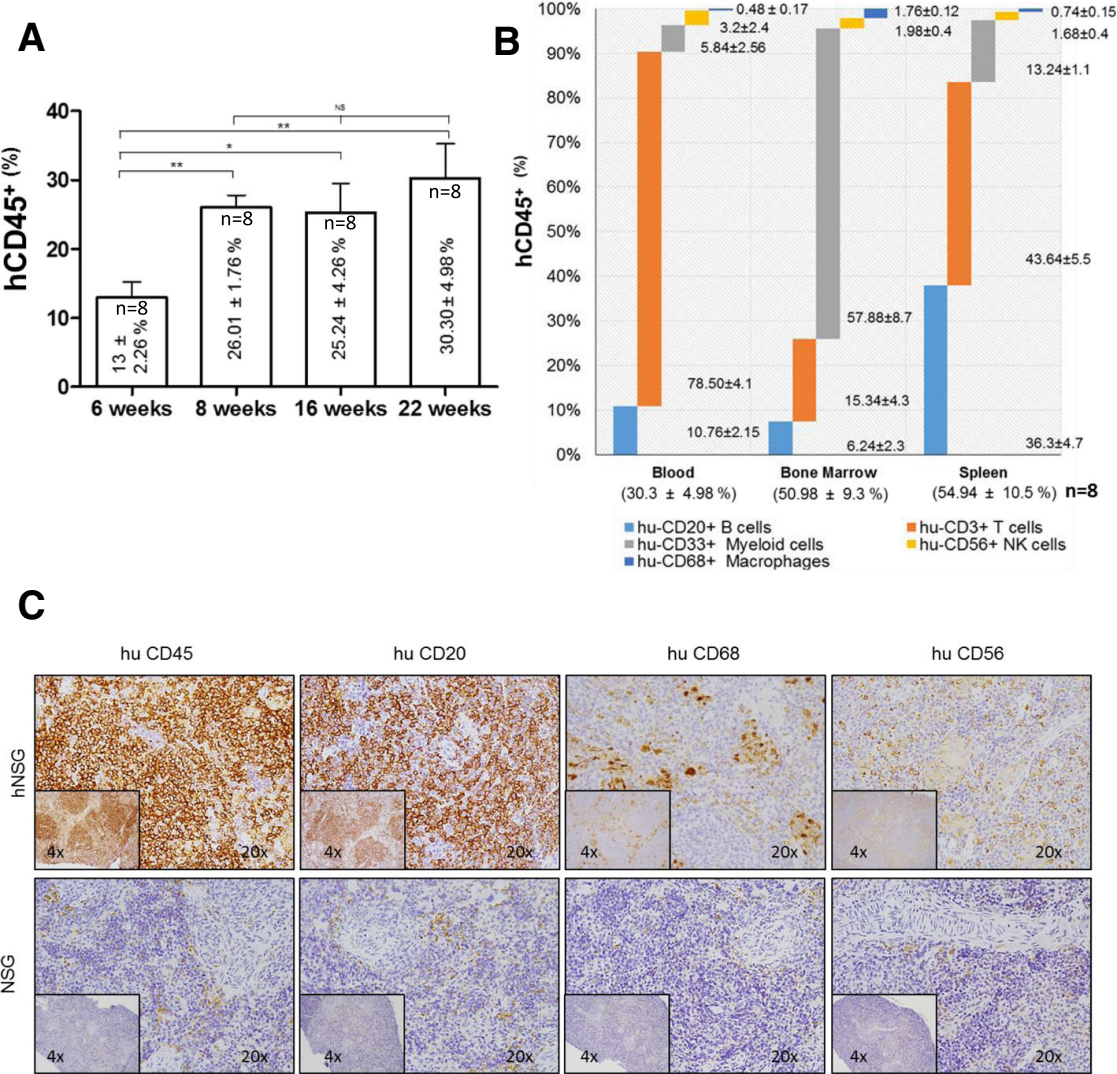
Analysis of human immune cell engraftment. **a** Evolution of the percentage of human CD45^+^ cells after intravenous (i.v.) injection of hCD34^+^ hematopoietic stem cells. Cells were identified by flow cytometry in circulating blood collected from humanized mice at the indicated time intervals (*n* = 8). **b** Analysis of hCD45^+^ and corresponding subpopulations, including hCD20^+^ (B cells), hCD3^+^ (T cells), hCD33^+^ (myeloid lineage), hCD56^+^ (natural killer [NK] cells), and hCD68^+^ (macrophages) cells, was determined by flow cytometry in blood, bone marrow, and spleen samples collected from humanized nonobese diabetic/severe combined immunodeficiency *IL2R*γ^*null*^ (hNSG) mice after 22 weeks of i.v. injection of human hematopoietic stem cells (*n* = 8). **c** Representative IHC analysis of human CD45^+^, CD20^+^, CD68^+^, and CD56^+^ cells performed in preparations of spleen from humanized (upper row) and nonhumanized (lower row) NSG mice. Counterstain, hematoxylin; magnifications, 20× and 4× (inset)

As mentioned above, one of the major limitations of preclinical studies with immunotherapies in breast cancer is the lack of availability of appropriate experimental models. Although human CD34^+^ HSC-engrafted NSG (hNSG) mice harboring different types of PDXs are commercially available, the high costs of these animal models limit, to some extent, their use by academic research groups. We have developed in-house established humanized mouse models that were generated by i.v. injection of hCD34^+^ HSCs as per protocols previously described [28, 29]. Briefly, 3–4-week-old NSG mice received a low, sublethal dose of irradiation, followed after 4 hours by tail vein injection of CD34^+^ HSCs. The presence of human cells was evaluated in blood collected from these animals at different time intervals starting at 6 weeks after the i.v. administration of hCD34^+^ HSC cells. The percentage of HSC engraftment was ~ 90% (on average) per group of mice injected (~ 80–100 mice/group). In agreement with multiple previous reports [29, 33, 34], the presence in blood of human CD45^+^ cells was readily detectable by week 6 (mean, 13 ± 2.26%), reaching percentages ~ 25% by weeks 8–16 (26.01 ± 1.76% and 25.24 ± 4.26%, respectively) and up to ~ 30% at week 22 (30.3 ± 4.98%) (Fig. 1a and Additional file 1: Figure S1). Analysis of hCD45^+^ subpopulations of cells, evaluated at week 22, showed the following distribution (expressed as percentage of hCD45^+^): hCD20^+^ (B cells), 10.76 ± 2.15%; hCD3^+^ (T cells), 78.5 ± 4.09%; hCD33^+^ (myeloid cells), 5.84 ± 5.26%; hCD56^+^ (natural killer [NK] cells), 3.2 ± 2.36%; and hCD68^+^ (macrophages), 0.48 ± 0.17% (Fig. 1b). The composition of human cell populations was also analyzed in cells collected from bone marrow and spleen, where levels of hCD45^+^ represented 50.98 ± 9.27% and 54.94 ± 10.53%, respectively. Additional details showing cell lineage distribution are depicted in Fig. 1b. IHC analysis was performed in samples from spleens of both humanized and nonhumanized NSG mice using an anti-hCD45 antibody, showing a robust presence of these cells only in hNSG mice (Fig. 1c, upper panels). Additional characterization of human cells showed expression of markers corresponding to B cells (hCD20^+^), macrophages/myeloid lineage (hCD68^+^), and NK cells (hCD56^+^). Importantly, none of the human markers were detected in samples from non-hNSG, confirming the specificity and level of humanization achieved in hNSG mice (Fig. 1c, bottom panels).

### Breast cancer tumor transplant and development in hNSG mice

**Fig. 2:**
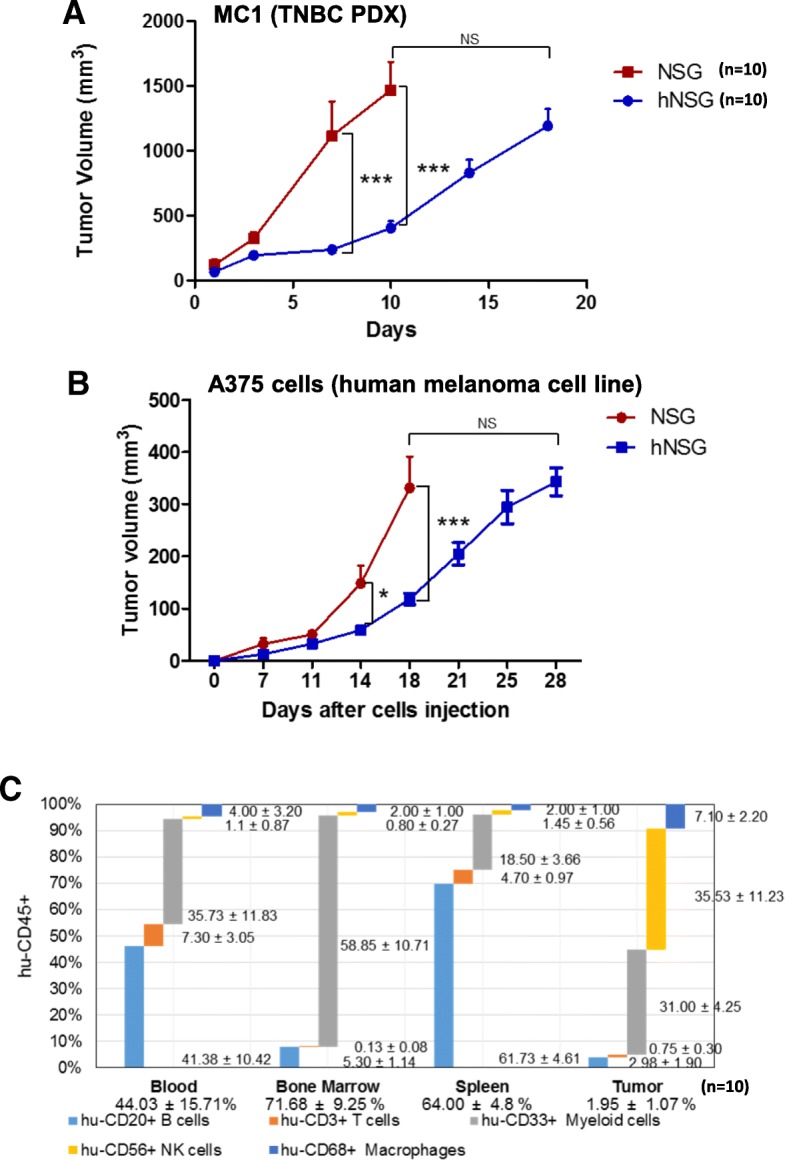
In vivo effects of humanization of nonobese diabetic/severe combined immunodeficiency *IL2R*γ^*null*^ (NSG) mice in the growth and engraftment of triple-negative breast cancer (TNBC) patient-derived xenograft (PDX) tumor line MC1 (**a**) and human melanoma A375 cell line (**b**). Both humanized and nonhumanized female NSG mice (*n* = 10 in each group) were transplanted orthotopically with pieces of either the PDX tumor line MC1 (into the cleared mammary fat pad) or A375 cells (into the skin) and allowed to grow. Tumor volume was determined twice weekly. *NS* Nonsignificant; *P < 0.05, *** *P* < 0.001. **c** Flow cytometric analysis of human CD45^+^ cells and hCD20^+^ (B cells), hCD3^+^ (T cells), hCD33^+^ (myeloid lineage), hCD56^+^ (natural killer [NK] cells) and hCD68^+^ (macrophages) cell subpopulations determined in blood, spleen, bone marrow, and MC1 PDX tumors of the corresponding samples shown in (**a**) (*n* = 10)

In order to develop and establish the appropriate mouse models to test immunotherapies against TNBC, we next directed our efforts toward obtaining PDX models harbored in the hNSG mice. To this end, we used patient-derived breast cancer tumor lines from our existing collection, previously established in immune-compromised SCID/beige mice [12]. Low-passage fresh xenograft tumor fragments of the breast cancer line MC1 [30] were transplanted into the cleared mammary gland fat pad of recipient nonhumanized and humanized NSG mice. Tumor volume was then evaluated over time. Approximately 80–85% positive tumor engraftment was observed, slightly lower than what is normally achieved in nonhumanized mice (i.e., ~ 95–100% under the same experimental conditions). As depicted in Fig. 2, after the tumors were palpable (~ 100–150 mm^3^; day 0), fast and aggressive tumor growth was observed in non-hNSG mice, reaching the maximum humane size before killing by day 10. In the case of hNSG mice, the growth of MC1 tumors was slower, achieving a similar volume only after day 18. To further characterize the hNSG model, A375 melanoma cell xenografts were grown in both nonhumanized and humanized NSG mice. As was the case with TNBC PDXs, melanoma cell xenograft growth also appeared to be delayed in hNSG animals when compared with nonhumanized NSG mice (Fig. 2b), highlighting the potential role of humanization and acquisition of a competent immunological status in affecting the growth of a tumor [35], as previously shown in similar models [36, 37]. To further investigate these observations, human leukocyte antigen (HLA) subtyping was performed in both the original hCD34^+^ HSCs and two of the PDXs used in this study by using standard protocols used at the Department of Pathology & Genomic Medicine, Immunobiology & Transplant Science Center, Houston Methodist Hospital (Houston, TX, USA). Both PDX tumor models displayed different HLA subtypes (Additional file 2: Table S1), whereas the analysis of hCD34^+^ HSCs resulted in the possibility of multiple patterns consistent with a mix of HLA types, which did not allow for a specific identification. These results are consistent with the fact that the hCD34^+^ HSCs (STEMCELL Technologies) used in this study are basically formed by a pool of cells from different donors. This situation of partially matched HLA typing between hNSG mice and the PDXs may have contributed to lower tumor immunogenic rejection while simultaneously resulting in reduced percentages of engraftment and slower growing tumors (Fig. 2), as previously observed in similar studies showing that human PDX tumors can grow in hNSG with partially HLA-matched allogeneic human immune systems [36, 37].

Analysis of hCD45^+^ cells in blood, spleen, and bone marrow, performed at the moment the tumors reached their maximum size, showed profiles similar to those observed in animals not harboring tumors (i.e., hCD45^+^, 44.03 ± 15.71, 71.68 ± 9.25, and 64.00 ± 4.8 for blood, bone marrow, and spleen, respectively). A detailed distribution of the different CD45^+^ subpopulations is displayed in Fig. 2c, including the corresponding TILs isolated from the tumors (hCD45^+^, 1.95 ± 1.07).

To further characterize the humanized PDX model, levels of human cytokines known to be involved in the response to immunomodulatory therapies were determined in samples of serum and tumor lysates collected from nonhumanized NSG and hNSG mice harboring PDXs (Table 1) [38, 39]. As expected, significant increases were found in both circulating and tumor contents in the humanized mice. Importantly, taking into account the species specificity of the antibodies included in the assay, the presence of some circulating human cytokines detected in the nonhumanized NSG mice (e.g., granulocyte-macrophage colony-stimulating factor [GM-CSF], interleukin [IL]-6, and IL-8) were considered to have originated from the PDX because their levels, which were among the highest of the panel, were also clearly detected in the tumor collected from nonhumanized NSG mice. One of the recognized limitations of the hNSG mouse model resides in the absence of key cytokines that may support the stable engraftment of myeloid lineages, notably GM-CSF [40]. Interestingly, as the present results show, PDX-mediated production of GM-CSF may have contributed to this situation, as clearly evidenced by the fact that, despite the total levels of hCD45^+^ cells being similar between hNSG mice with/without PDXs, the percentage of the myeloid lineage subpopulation, represented by hCD33^+^ cells, was significantly increased in those mice harboring the tumors (Fig. 2c). Consequently, this may have resulted in a better reconstitution of the human immune system in the blood and thereby improved the accuracy of the studies that were performed with them.

**Table 1:** Levels of specific human cytokines

Human cytokines				
	Serum		Tumor	
	Non-hNSG	hNSG	Non-hNSG	hNSG
IL-1B	0	0	5.3 ± 1.0	10.9 ± 0.8*
TNF-α	0	1.3 ± 0.5	25.4 ± 2.1	250.2 ± 35.4**
IL-5	0	1.5 ± 0.4	0	0
IL-2	0	1.8 ± 0.3	0	0
IL-7	0	2.1 ± 0.6	10.4 ± 0.2	28.4 ± 0.6**
IL-12	0	3.6 ± 0.3	0	0
IFN-γ	0	19.8 ± 5.4	0	11.75 ± 0.6**
IL-13	0	0	7.8 ± 0.5	16.60 ± 0.6**
IL-4	0	0	8.3 ± 0.4	21.40 ± 1.0**
GM-CSF	93.7 ± 5.6	94.8 ± 9.7	841.8 ± 93.9	3296.3 ± 235.2**
IL-6	45.1 ± 2.5	73.3 ± 2.6*	217.8 ± 12.5	1039.2 ± 100.4**
IL-8	2989.5 ± 527.8	2798.3 ± 503.9	1208.1 ± 114.9	1310 ± 61.7

*Abbreviations: GM-CSF* Granulocyte-macrophage colony-stimulating factor, *hNSG* Humanized nonobese diabetic/severe combined immunodeficiency *IL2R*γ^*null*^, *IFN-γ* Interferon-γ, *IL* Interleukin, *TNF-α* Tumor necrosis factor-α

Cytokines were measured in samples of serum and tumor lysates of non-hNSG and hNSG mice harboring triple-negative breast cancer MC1 patient-derived xenografts. Values are expressed in picograms per milliliter (± SEM) (*n* = 4 NSG; *n* = 6 hNSG).*P < 0.05, **P < 0.01

**Fig. 3:**
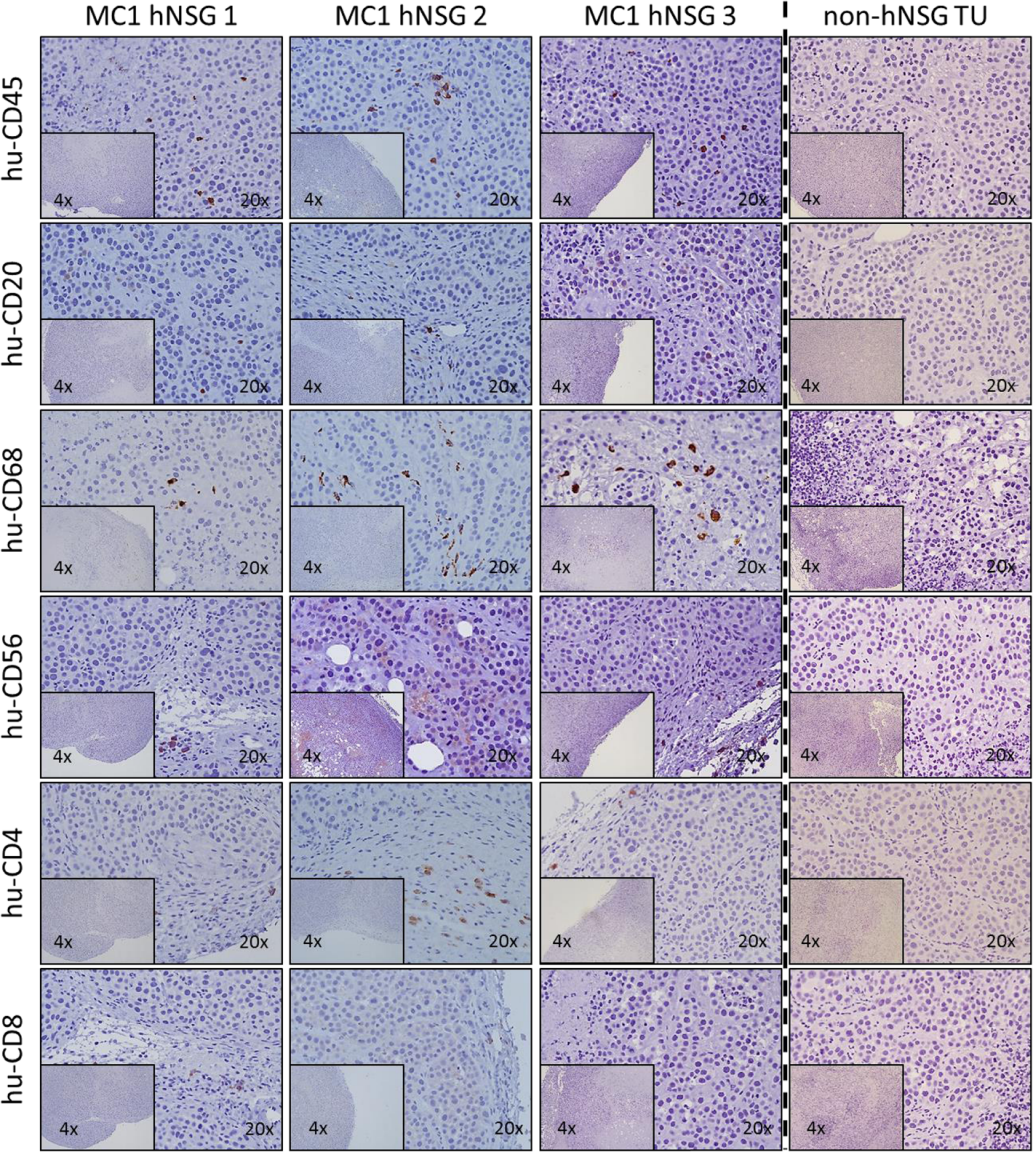
IHC analysis of human CD45^+^, CD20^+^, CD68^+^, CD56^+^, CD4^+^, and CD8^+^ cells and cells present in MC1 tumor xenografts. Representative images (from a total of 8–10 processed samples in each group) of IHC performed in preparations of MC1 tumor samples grown in either humanized or nonhumanized nonobese diabetic/severe combined immunodeficiency *IL2R*γ^*null*^ (NSG) mice corresponding to samples shown in Fig. 2a or c, respectively. 4× (inset) and 20× magnifications are shown; counterstain, hematoxylin.

IHC analysis was then performed on the tumors after they were collected. As shown in Fig. 3, the presence of hCD45^+^ cells was detectable in all the tumors screened (samples from different individual animals are shown), localizing both toward the periphery of the tumors as well as inside them. Analysis of hCD45^+^ cell subpopulations also showed hCD20^+^ cells (B cells), hCD68^+^ (macrophages), hCD56^+^ (NK cells), hCD4^+^ (T-helper cells), and hCD8^+^ T-cytotoxic cells. Importantly, the expression of human cell markers remained negative in MC1 tumors developed in nonhumanized NSG mice, indicating the specificity of the cells detected in the corresponding humanized MC1 tumor engraftments.

### Breast cancer metastasis to the lung in hNSG mice

One of the most relevant characteristics of PDX models is their ability to retain the morphology, cellular heterogeneity, and molecular profiles of the original patient tumors [11]. To determine whether the immunological condition of the host (i.e., non-hNSG vs. hNSG) may have altered the genetic profile of the tumors, gene expression analysis of MC1, BCM-2147, and BCM-4913 PDXs growing in either non-hNSG or hNSG mice was performed by RNA sequencing (RNA-seq). Importantly, only minimal differences in the number of genes differentially expressed were found, demonstrating that the immunological status of the host played no significant role in the genetic stability of the tumors during the time course of the study (Additional file 2: Table S2).

**Fig. 4:**
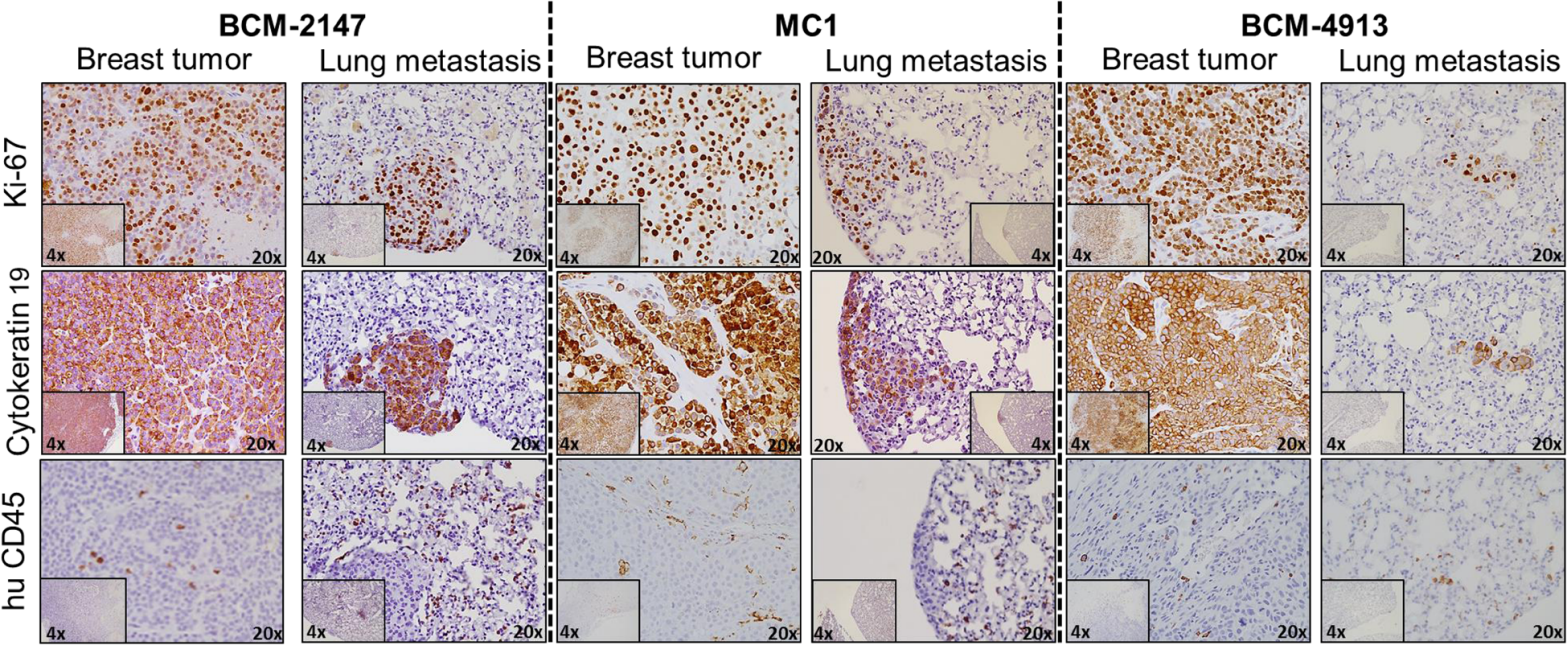
Analysis of breast cancer lung metastasis in humanized nonobese diabetic/severe combined immunodeficiency *IL2R*γ^*null*^ (hNSG) patient-derived xenograft (PDX). IHC analysis of human Ki-67, cytokeratin 19, and CD45^+^ expression in primary (breast) and metastatic (lung) triple-negative breast cancer PDX tumor lines BCM-2147, MC1, and BCM-4913 engrafted in hNSG mice. Amplifications, 4× and 20×; counterstain, hematoxylin

Orthotopic breast cancer transplant models have been shown to recapitulate the same metastatic lesions and sites [11]. To determine whether the metastatic characteristics were maintained in the hNSG mouse model, PDXs corresponding to TNBC MC1, BCM-2147, and BCM-4913 tumor lines, all of which are known to produce metastatic lesions to the lung, were analyzed [12]. PDXs were transplanted into the cleared mammary gland fat pad of hNSG mice as described in the Methods section. At the moment of tumor removal, mice were checked for the appearance of metastasis in the lungs. As shown in Fig. 4 (representative results of each tumor line are shown; not all the animals analyzed displayed lung metastasis), IHC performed in the primary breast tumor showed expression of the human proliferation marker Ki-67 and the breast cancer marker CK19, confirming the human nature of the primary PDX. Importantly, as previously described in models using the MC1 tumor (Fig. 3), the presence of hCD45^+^ cells was detectable in all three primary tumor lines (Fig. 4). IHC assays using Ki-67 and CK19 identified the lung metastatic microscopic regions corresponding to the tumor localization (Fig. 4). As in the primary breast tumor, the presence of hCD45^+^ cells was also observed in both the lung and the proximities of the metastatic tumor (Fig. 4). Analyses of hCD45^+^ subpopulations in lung and lung metastasis, including hCD4, hCD3, hCD8, hCD20, hCD68, and hCD56, were also performed by IHC (Additional file 3: Figure S2). Together, these results demonstrate that one of the main characteristics of the TNBC PDXs (i.e., their capability to metastasize to the lungs) remains conserved in humanized mouse models.

### Expression of PD-L1 in TNBC PDXs

**Fig. 5:**
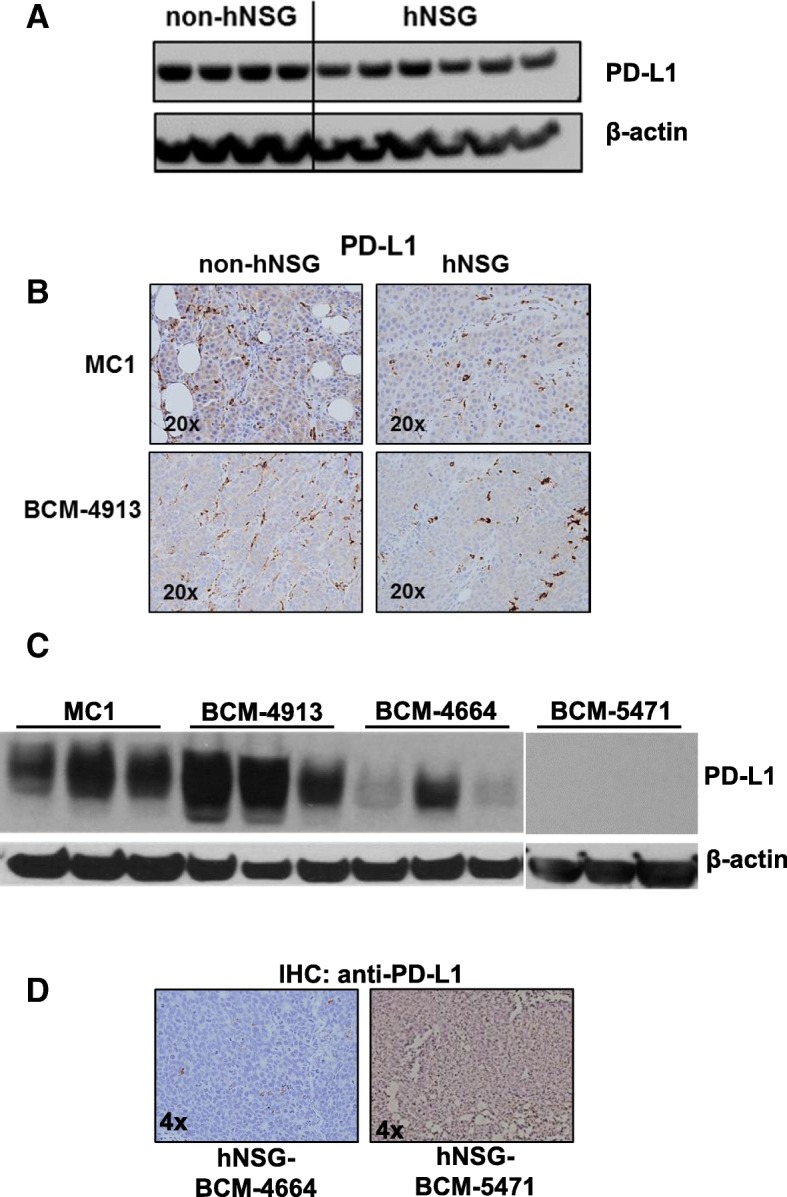
Analysis of programmed death ligand 1 (PD-L1) protein expression in patient-derived xenograft (PDX) tumor samples engrafted in both nonhumanized and humanized nonobese diabetic/severe combined immunodeficiency *IL2R*γ^*null*^ (hNSG) mice performed by Western blotting (**a**, MC1) or IHC (**b**, upper panels, MC1; lower panels, BCM-4913). In Western blotting experiments, samples were blotted with an anti-β-actin antibody as a loading control. The blots were processed in parallel, and they were all sourced from the same experiment. **c** Comparative analysis of PD-L1 levels was performed using four different PDX tumor lines (MC1, BCM-4913, BCM-4664, BCM-5471) engrafted in hNSG mice. Three independent tumors (animals) of each PDX line were evaluated by Western blot analysis. Samples were blotted with an anti-β-actin antibody as a loading control. **d** PD-L1 analysis performed by IHC of BCM-4664 and BCM-5471 PDXs engrafted in hNSG mice. 4× magnifications are shown; counterstain, hematoxylin

Although still under continuous evaluation, both the expression of PD-L1 and a high mutational load have been associated with response to immune CPIs in clinical trials evaluating the efficacy of anti-PD-1-based therapies in melanoma, lung cancer, and TNBC [41–45]. The expression of PD-L1 was then determined in cell lysates of several PDX tumor lines by both Western blotting and IHC. As shown in Fig. 5a, a robust expression of PD-L1 was observed in MC1 PDXs collected from both non-hNSG and hNSG mice. Furthermore, this expression was not affected by the immunological status (i.e., humanized or nonhumanized) of the mice. Similarly, strong expression was also observed in PDX BCM-4913, as determined by both Western blotting and IHC (Fig. 5b and c). However, individual samples from two additional PDX tumor lines, BCM-4664 and BCM-5471, displayed significantly lower expression of PD-L1 (Fig. 5c and d, Western blot and IHC, respectively). Together, these results provide evidence showing the variability of PD-L1 expression over different TNBC PDXs, recapitulating the situation often found in the clinical field [46].

### Effects of anti-PD-1 therapy in the treatment of TNBC PDXs

**Fig. 6:**
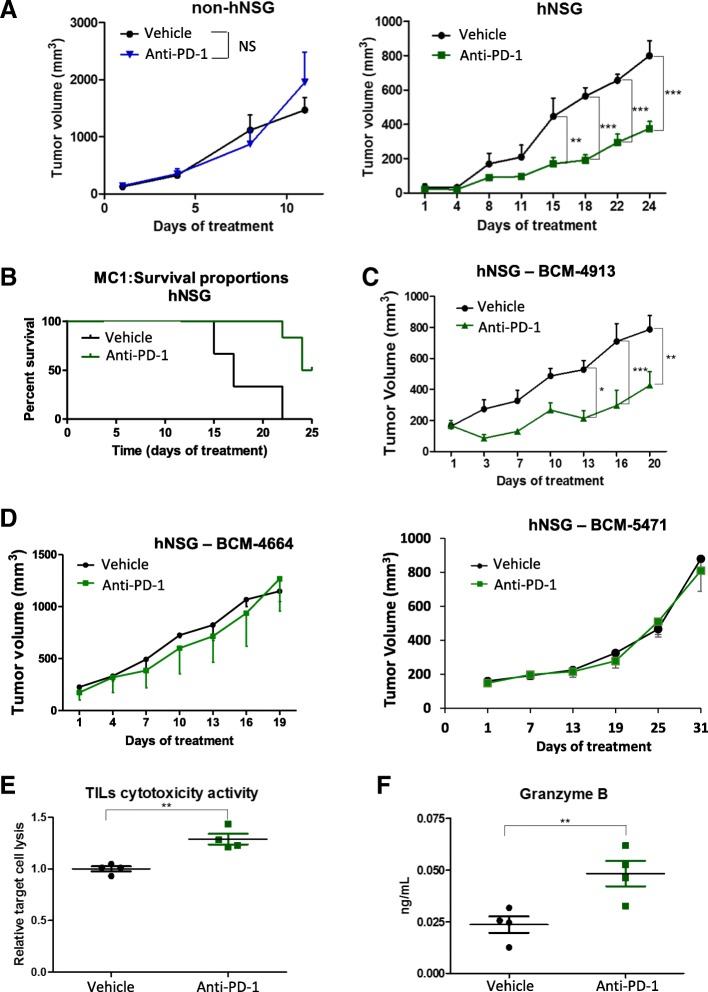
Response of triple-negative breast cancer (TNBC) patient-derived xenografts (PDXs) to the anti-programmed cell death protein 1 (anti-PD-1) therapy. **a** In vivo treatment with anti-PD-1 antibody (10 mg/kg intravenous [i.v.] once weekly) of either TNBC MC1 PDX-engrafted nonhumanized (left graph, *n* = 5) or humanized (right graph, *n* = 5) nonobese diabetic/severe combined immunodeficiency *IL2R*γ^*null*^ (hNSG) mice. Tumor volume was measured twice weekly. **b** Kaplan-Meier analysis of median survival of mice treated with vehicle (*n* = 6) vs. anti-PD-1 antibody (*n* = 6). **c** hNSG mice engrafted with an additional TNBC BCM-4913 PDX tumor line were treated with either vehicle control or anti-PD-1 antibody (10 mg/kg i.v. once weekly). Tumor volumes were measured twice weekly. **d** In vivo treatment with anti-PD-1 antibody (10 mg/kg i.v. once weekly) of TNBC BCM-4664 (*n* = 5) and HM-3818 (*n* = 5) PDXs engrafted in hNSG mice. Tumor volume was measured twice weekly. **e** Analysis of tumor-infiltrating lymphocyte (TIL) cytotoxic activity. TILs isolated by Ficoll gradient from vehicle- or anti-PD-1 antibody-treated MC1 PDX tumors engrafted in hNSG mice were cocultured with disaggregated MC1 tumor cells obtained from the corresponding PDX grown in nonhumanized NSG mice. Cytotoxic activity was measured using the CytoTox 96® Non-Radioactive Cytotoxicity Assay as per the manufacturer’s instructions. **f** Levels of granzyme B tumor were measured by incubating tumor protein lysates with antibody-immobilized magnetic beads and evaluated using a Luminex LX200 Multiplexing Assay System. **P < 0.01, ***P < 0.001. *NS* Nonsignificant

Next, the efficacy of an anti-PD-1-based therapy was evaluated in our established hNSG PDX models. First, both non-hNSG and hNSG mice were implanted with MC1 PDXs and treated following a weekly schedule of humanized anti-PD-1 (10 mg/kg i.v.). As depicted in Fig. 6a (left graph), administration of anti-PD-1 antibody (nivolumab) to non-hNSG mice had no effect on the tumor size and growth, because tumors in both vehicle- and anti-PD-1-treated animals reached similar volume after days 10–12 of therapy (corresponding to two cycles of i.v. administered anti-PD-1 antibody). However, when the same schedule was applied to MC1-harboring hNSG animals, a significant reduction in the rate of MC1 tumor growth/volume was observed in the group of anti-PD-1-treated animals (Fig. 6a, right graph). In agreement with these results, analysis of survival rates, with endpoint based on the time that animals needed to be killed because of the tumor size, showed improved survival in the anti-PD-1-treated group vs. the corresponding vehicle-treated controls (Fig. 6b). The anti PD-1 monotherapy was then tested in additional TNBC PDX tumor lines. hNSG mice harboring the BCM-4913 PDXs were treated with pembrolizumab (10 mg/kg), following the same schedule used with the MC1 PDXs (i.e., weekly i.v. injections), resulting also in a significant reduction in tumor growth (Fig. 6c). Importantly, and consistent with the results observed in clinical settings showing despair activity of anti-PD-1/PD-L1 therapies in TNBC tumors [47–49], anti-PD-1 treatment resulted ineffective in two additional PDX models, BCM-4664 and BCM-5471 (Fig. 6d).

In addition, the effects of ipilimumab, a U.S. Food and Drug Administration-approved immune CPI directed against CTLA-4, were also evaluated for efficacy against MC1 PDXs. Once tumors reached ~ 150 mm^3^, animals were treated weekly with 10 mg/kg i.v. injections for up to 3 weeks. In contrast to the anti-PD-1-based therapies and in line with previous reports on breast cancer [50, 51], anti-CTLA-4 monotherapy did not result in a therapeutic benefit in MC1 PDXs (Additional file 4: Figure S3).

To identify potential mechanisms of action involved in the anti-PD-1-mediated TNBC tumor growth inhibition, the amount of TILs present in MC1 PDX tumors collected from both vehicle- and anti-PD-1-treated animals was determined by flow cytometry. Interestingly, no significant differences were observed in the percentage of human immunological cells infiltrating the tumor tissue (Additional file 5: Figure S4A). We then evaluated the cytotoxic activity of TILs by measuring the levels of lactate dehydrogenase, a stable cytosolic enzyme that is released upon TIL-induced tumor cell lysis. The experimental setting is described in the Methods section and in Additional file 5: Figure S4B. Briefly, TILs from MC1 PDX tumors engrafted in hNSG mice treated with either vehicle or anti-PD-1 antibody were isolated and then cocultured with disaggregated MC1 tumor cells obtained from the corresponding PDX grown in nonhumanized NSG mice. As shown in Fig. 6e, TILs corresponding to mice treated with the anti-PD-1 antibody displayed significantly higher cytotoxic activity than those corresponding to mice treated with vehicle control. Consistently, levels of granzyme B, a serine protease found in and released by TILs, were also significantly higher in lysates from tumors treated with anti-PD-1 than in those from vehicle-treated control lysates (Fig. 6f). In line with these findings, it is noteworthy that levels of IFN-γ, a cytokine secreted by activated T cells [52], was detected only in both serum and tumor lysates of PDX-harboring hNSG mice, indicating that it may have originated from human cytotoxic lymphocytes in response to the presence of PDXs. Together, these observations suggest that treatment with the anti-PD-1 resulted in increased cytotoxic activity of TILs present in the TNBC PDX tumors rather than in a higher number of TILs locating in the tumor tissue.

**Fig. 7:**
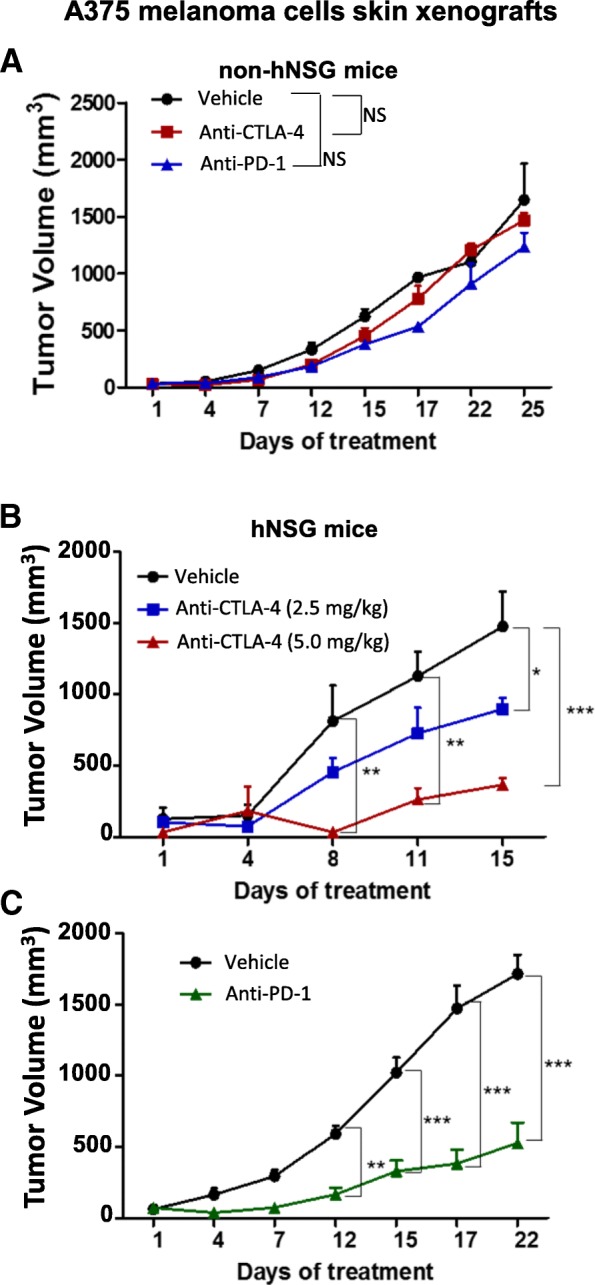
Analysis of A375 melanoma cell line xenograft growth. Human melanoma cells (A375; 5 × 10^5^) were injected orthotopically into the skin of both nonhumanized nonobese diabetic/severe combined immunodeficiency *IL2R*γ^*null*^ (NSG) and humanized NSG (hNSG) mice, after which (initial tumor volume 150–200 mm^3^) they were randomly sorted into treatment groups. Non-hNSG mice (**a**) or hNSG mice (**b** and **c**) were treated weekly with vehicle (control), anti-CTL4 (2.5/5 mg/kg (**b**), or anti-PD-1 (10 mg/kg) (**c**) antibodies. Tumor growth was evaluated twice weekly. If tumor volume reached 1500–2000 mm^3^, mice were killed as per humane animal welfare regulations. *P < 0.05, **P < 0.01, *** *P* < 0.001. *NS* Nonsignificant

To further characterize and validate our humanized mouse models and their use in immunotherapy-targeted preclinical studies, similar studies were performed by generating xenografts with the immunogenic A375 melanoma cell line implanted orthotopically into the skin of both non-hNSG and hNSG mice (Fig. 7). As previously shown with MC1 TNBC PDXs (Fig. 6a), treatment with either anti-CTLA-4 or anti-PD-1 antibodies had no effect on the progression of melanoma tumors implanted in non-hNSG mice (Fig. 7a). However, consistent with previous clinical studies [3, 53, 54] and its highly immunogenic profile, both anti-CTLA-4 and anti-PD-1 antibodies were highly effective in suppressing the growth of the melanoma cell xenografts (Fig. 7b and c), including a significant dose-dependent response with anti-CTLA-4 therapy (Fig. 7b). These results provide additional evidence of both the humanization of the NSG model used and the relevance that such a model may have for testing immunotherapy-based regimens.

## Discussion

The use of immunotherapies in breast cancer has been limited by breast cancer’s relatively low immunogenicity [55]. However, newly developed strategies and/or approaches are rapidly changing the field, and novel immune CPIs are already approved or under different phases of clinical evaluation. Examples of these studies include clinical evaluation of anti-PD-1 and anti-PD-L1 therapies, administered either as single drugs or as part of multiple combinations [56, 57]. Enrichment strategies to select for patients more likely to respond have identified the expression and testing of PD-L1 to be a potentially useful predictive marker in guiding this process [58–60]. Following these criteria, in the present study, we investigated the expression of PD-L1 and its correlation with the anti-PD-1 activity. Although we did not evaluate a number of PDX tumor lines large enough to have the power required to achieve a statistically supported conclusion, our results showed a trend: Those PDXs that expressed high levels of PD-L1 appeared to respond to the anti-PD-1 therapy. Several clinical studies have evaluated the expression of PD-L1 and tried to identify possible associations with the therapeutic response. For example, positive expression of PD-L1 in TNBC stromal tissue or in ≥ 1% of tumor cells has been used as a potential predictive biomarker in the phase Ib KEYNOTE-012 clinical trial [47]. Here, an 18.5% overall response rate was observed in the PD-L1-positive group, which represented ~ 60% of the total number of heavily pretreated patients with advanced TNBC under evaluation [47]. Other studies included a retrospective analysis (between 2004 and 2013) of 136 TNBC cases without neoadjuvant therapy, showing that stromal PD-L1 expression was significantly associated with better disease-free survival (DFS), whereas no association was found between PD-1 expression and DFS, overall survival, or metastasis [61]. Additional observations made by Botti et al. also showed a strong association between PD-L1 expression and better DFS [62]. Similar outcomes have resulted from a phase Ia study of the anti-PD-L1 antibody atezolizumab in previously treated patients with TNBC [63], altogether adding supporting evidence to the notion that PD-L1 expression may represent an important biomarker for prognostic stratification and CPI-based therapies. Nonetheless, the current consensus is that in addition to the expression of PD-L1 and mutation burden, multiple biomarkers may be needed to determine which patients will likely benefit from immunotherapies, including, notably in TNBC and HER2-positive patients, the presence of CD8^+^ TILs, immune-related gene signatures, and multiplex IHC assays that may take into account the pharmacodynamic and spatial interactions of the TME [55, 56, 64–66]. As we demonstrated in the present study, our hNSG PDX model displayed clear evidence of several of these parameters (i.e., a humanized immune system with detectable presence of hCD45^+^ TILs and cytokine levels) and robust expression of PD-L1 in some of the tumor lines. These results are in line with the clinical studies previously mentioned where the therapeutic benefits of regimens containing immunomodulatory CPI were observed mainly in patients where both TILs and PD-L1 were present, which provides additional support for the use of the humanized TNBC PDX mouse model used in this work. Similarly, also in agreement with observations in clinical trials [51, 67], the present model showed limited or no activity when TNBC tumor line MC1 was treated with an anti-CTLA-4 antibody, further validating the humanized mouse model because it reproduces some of the most relevant results observed during the clinical evaluation of immune CPIs. In fact, anti-CTLA-4 monotherapies have shown no or very limited therapeutic advantage against breast cancer when administered alone [67], although their efficacy has been improved by combination with other agents [50, 51, 68], which opens the field to new investigations. The mechanisms leading to the apparent lack of anti-CTLA-4 activity when administered as a monotherapy in certain solid tumors, including breast cancer, are still not well understood. However, it is thought to be associated with tumors’ low antigenicity and microenvironment conditions that may not favor immune recognition [65, 69, 70].

From a potential mechanistic point of view, our studies indicate that the effects of blocking PD-1/PD-L1 interactions, thereby improving the immunological response [7, 8], may have resulted from increased activation of TILs rather than changes in the number of cells infiltrating the tumor. These observations are consistent with the established mode of action of these compounds (i.e., interfering the immune-inhibitory effects of the PD-1/PD-L1 interactions) [71]. In addition, our results may also suggest that amelioration of the therapeutic efficacy of immune CPIs could be achieved by modifying the TME as a way to enhance their activity, and in fact, multiple ongoing studies at both our and other laboratories are currently addressing this hypothesis. In addition, further studies are being designed to determine the long-term effects of CPIs in terms of tumor growth inhibition and mechanisms of resistance, notably in comparison to established chemotherapies, because the present report spanned a relatively short time frame.

In terms of the animal model that we used in the present study, it is clear that although these animals represent a very useful tool, humanization of NSG mice may still pose some technical challenges and/or limitations. Notably, one of those well-recognized limiting factors is the lack of GM-CSF, important for the differentiation and maturation of the myeloid lineage [72]. To address this point, several newer, genetically modified NSG-based (The Jackson Laboratory) or NOG (NOD/Shi-*scid*/IL-2Rγ^null^)-based (Taconic Biosciences, Rensselaer, NY, USA) models are being developed, which, by expressing the human cytokines GM-CSF and IL-3 and human stem cell factor gene (*SCF*; also known as KIT ligand, *KITLG*), allow for better engraftment of HSCs and cell lineage differentiation [73]. In our case, it is important to note that some of these limitations appeared to be compensated by the presence of the TNBC PDX. Indeed, as our results show, PDXs were associated with the presence of several cytokines, including GM-CSF, which consequently may have played an important role in improving the levels of the myeloid lineage (hCD33^+^ cells) when compared with the hNSG mice not harboring tumors. These results suggest, as previously mentioned, that the simultaneous presence of the PDX during hHSC engraftment may have compensated for the lack of this and other factors, contributing to a better reconstitution of the immune system.

Another important factor that was considered in our study was the potential role of matching HLA typing between the hNSG host and the PDXs. Our observations showed some differences in the PDX growth rate based on whether the mice were humanized or not, most likely owing to the incipient presence of an active immune system. However, as also shown by others, including the case of commercially available humanized PDX models [36, 37], no signs of graft-versus-host reaction were found. Furthermore, on the basis of the fact that the HLA typing of HSCs did not conclusively demonstrate compatibility with more than one pattern, it is plausible to postulate that the slower growth of PDXs may have resulted from partially HLA-matched hNSG/PDX engraftment, which allowed a seemingly regular tumor engraftment. This is an important observation because the ideal situation (i.e., isolating HSCs from the same cancer patient whose PDX is being used) may prove extremely difficult to achieve in large-scale preclinical studies, because of both the patient condition and the time usually required for a PDX to be established [73]. Alternatively, the use of immunocompetent syngeneic mouse models represents a valid approach. However, this also has its own limitations, mostly in terms of the availability of tumor models, the specificity of drugs being tested, and the extrapolation of observations to human cases. Together, despite some of the factors mentioned above that should be taken into consideration whenever using humanized PDX mouse models, these models still represent very helpful and sophisticated tools for preclinical evaluation of immune-based therapies, notably as they become more available and improved animal versions are generated.

## Conclusions

In the present work, we evaluated the preclinical efficacy of anti-PD-1 therapies developed in humanized mouse models of TNBC PDXs. Our results in this study (1) indicate that breast cancer PDX models engrafted in hNSG mice represent a valuable tool to test for immune-based therapies, as demonstrated by the differential effects of the anti-PD-1 therapy in either nonhumanized or humanized NSG mice; and (2) highlight the validity of our methodology developed “in-house.”

## Additional files

Additional file 1: Representative figure showing the results of flow cytometric analysis of human cells collected from blood of nonhumanized and humanized NSG mice after 8, 16, and 22 weeks of intravenous injection of human CD34^+^ hematopoietic stem cells (HSCs). Procedures and antibodies used in these studies are described in the Methods section. (PPTX 722 kb)

Additional file 2: **Table S1** Analysis of HLA type in PDX BCM-2147/-4913 and CD34^+^ HSCs. HLA typing was performed by using PCR-SSO DNA-based procedures. The serological phenotype is an interpretation based on molecular typing data. *ND* Not determined. **Table S2** Gene expression analysis (RNA-Seq) comparing MC1, BCM-2147, and BCM-4913 PDXs growing in nonhumanized vs. humanized NSG mice. Differentially expressed genes (DEGs) were selected by edge R-based *p* value and fold change (FC). Supplemental Methods. (DOCX 22 kb)

Additional file 3: IHC analysis of human CD4-, CD3-, CD8-, CD20-, CD68-, CD4-, and CD8-positive cells present in BCM-2147, MC1, and BCM-4913 tumor xenograft lung micrometastases. Representative IHC images of obtained using preparations of tumor samples grown in humanized NSG mice; 4× and 20× magnifications are shown counterstained with hematoxylin. (PPTX 3007 kb)

Additional file 4: Effects of the anti-CTLA-4 immune checkpoint inhibitor antibody ipilimumab against MC1 PDXs implanted in hNSG mice. Once tumors reached ~ 150 mm^3^, animals were treated weekly with 10 mg/kg intravenous injections for up to 3 weeks; tumor volumes were evaluated twice weekly. The values represent the mean ± SEM (*n* = 8). (PPTX 50 kb)

Additional file 5: **a** Evaluation of the percentages of human CD45^+^ TILs present in MC1 PDX tumors engrafted in hNSG mice and collected from animals treated with either vehicle control or anti-PD-1 antibody. The values represent the mean ± SEM (*n* = 8). **b** Schematic representation of the method used to determine the cytotoxic activity of TILs by measuring the levels of the lactate dehydrogenase (LDH), a stable cytosolic enzyme that is released upon TIL-induced tumor cell lysis. TILs were isolated from MC1 PDX tumors engrafted in hNSG mice and treated with either vehicle or anti-PD1 antibody that were cocultured with disaggregated MC1 tumor cells obtained from the corresponding PDX grown in nonhumanized NSG mice. (PPTX 131 kb)

## Abbreviations

ANOVA: Analysis of variance
APC: Allophycocyanin
CK19: Cytokeratin 19
CPI: Checkpoint inhibitor
CTLA-4: Cytotoxic T-lymphocyte-associated protein 4
DFS: Disease-free survival
FITC: Fluorescein isothiocyanate
GM-CSF: Granulocyte-macrophage colony-stimulating factor
HLA: Human leukocyte antigen
hNSG: Humanized nonobese diabetic/severe combined immunodeficiency *IL2R*γ^*null*^
HSC: Hematopoietic stem cell
IgG: Immunoglobulin G
IL: Interleukin
i.v.: Intravenous(ly)
NK: Natural killer cells
PD-1: Programmed cell death protein 1
PD-L1: Programmed death ligand 1
PDX: Patient-derived xenograft
PE: Phycoerythrin
RNA-seq: RNA sequencing
TIL: Tumor-infiltrating lymphocyte
TME: Tumor microenvironment
TNBC: Triple-negative breast cancer
