# Supplementary figures and images for: Evaluation of anti-PD-1-based therapy against triple-negative breast cancer patient-derived xenograft tumors engrafted in humanized mouse models
Source: Breast Cancer Res. 2018 Sep 5;20:108. doi: 10.1186/s13058-018-1037-4 (PMC6125882; doi:10.1186/s13058-018-1037-4)

## Slide 1
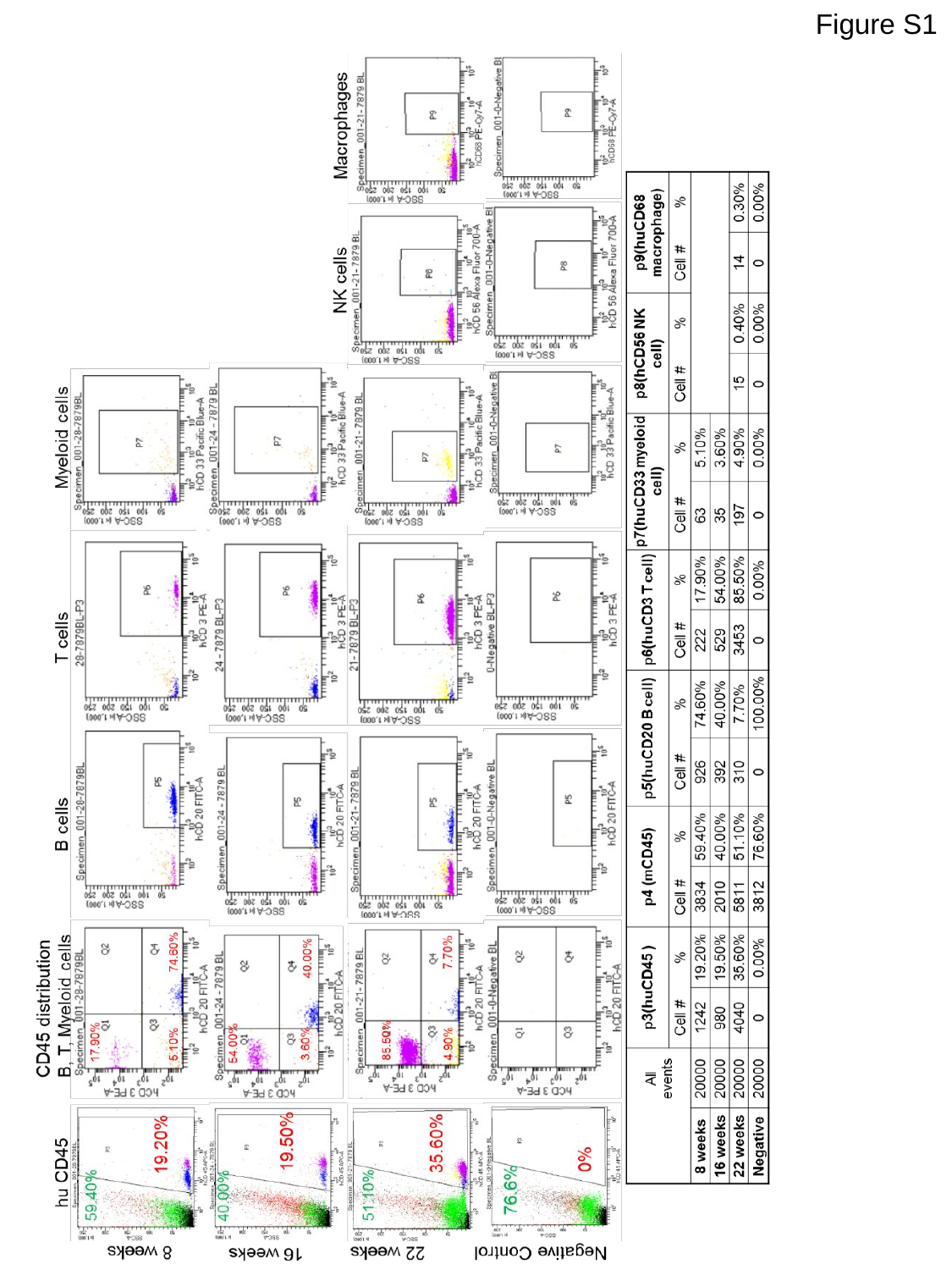

Figure S1

Supplement: Supplementary file 1 — Representative figure showing the results of flow cytometric analysis of human cells collected from blood of nonhumanized and humanized NSG mice after 8, 16, and 22 weeks of intravenous injection of human CD34+ hematopoietic stem cells (HSCs). Procedures and antibodies used in these studies are described in the Methods section. (PPTX 722 kb) [file 13058_2018_1037_MOESM1_ESM.pptx]

## Slide 1
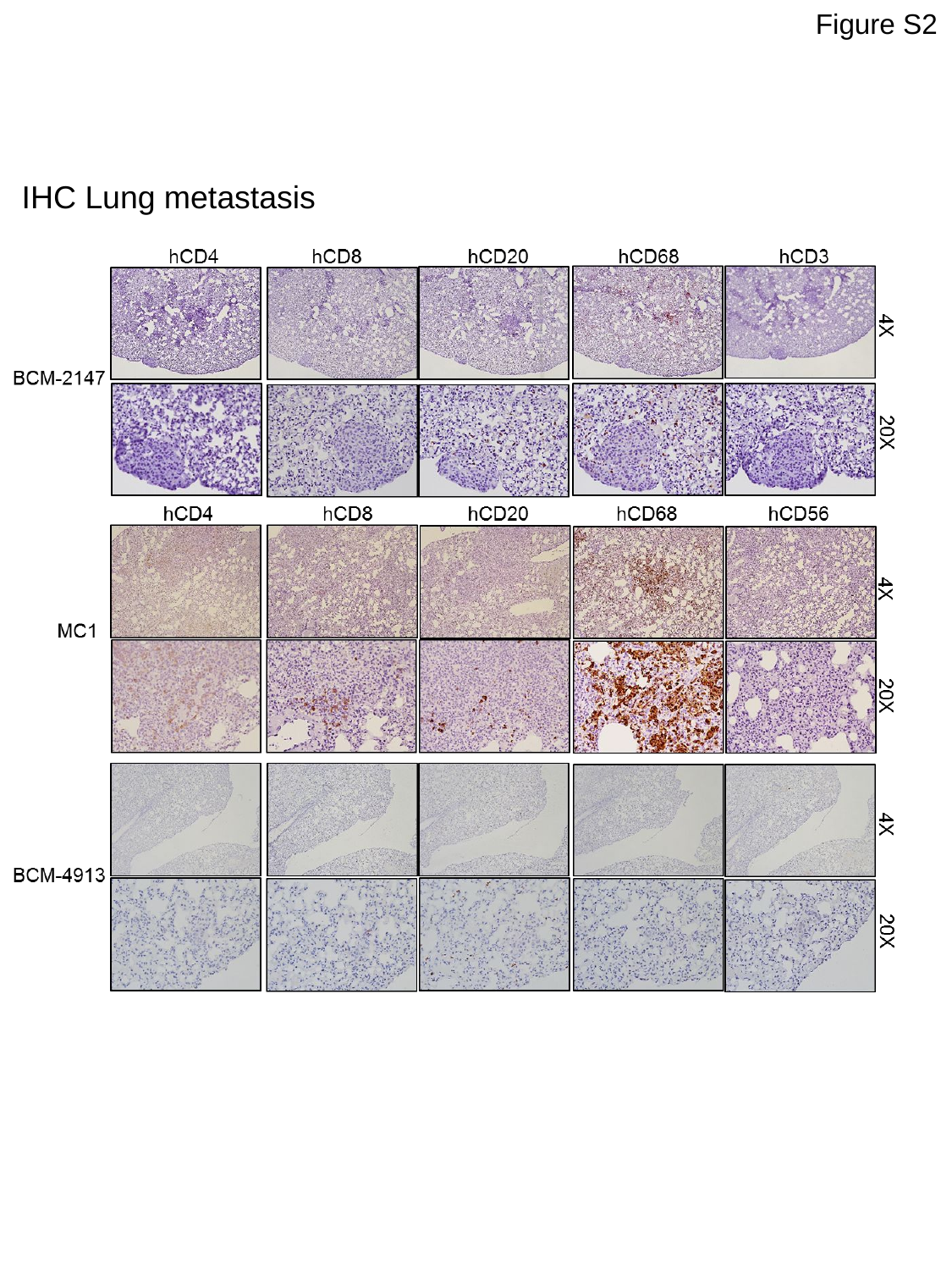

Figure S2
IHC Lung metastasis

Supplement: Supplementary file 3 — IHC analysis of human CD4-, CD3-, CD8-, CD20-, CD68-, CD4-, and CD8-positive cells present in BCM-2147, MC1, and BCM-4913 tumor xenograft lung micrometastases. Representative IHC images of obtained using preparations of tumor samples grown in humanized NSG mice; 4× and 20× magnifications are shown counterstained with hematoxylin. (PPTX 3007 kb) [file 13058_2018_1037_MOESM3_ESM.pptx]

## Slide 1
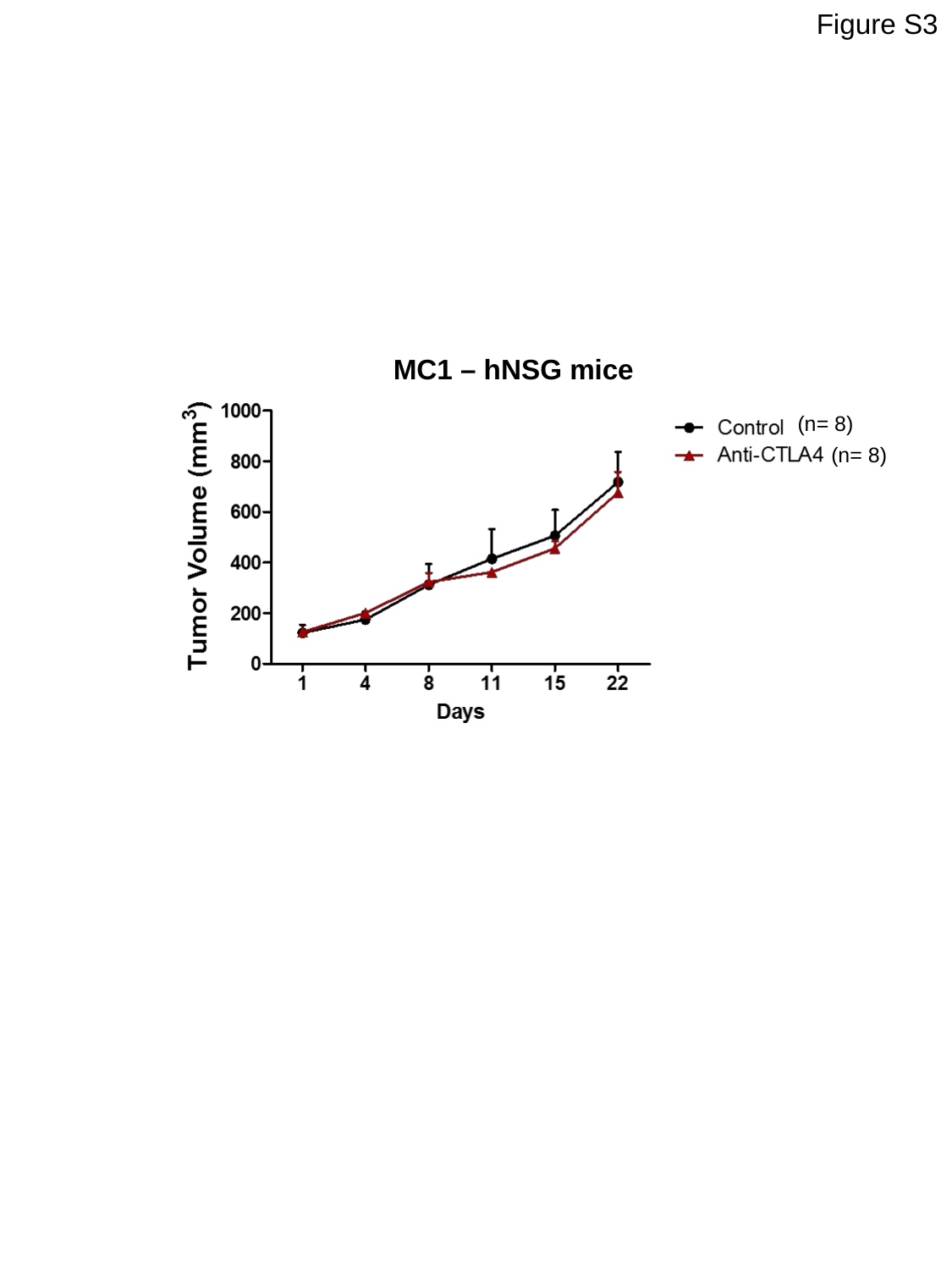

Figure S3
MC1 – hNSG mice
(n= 8)
(n= 8)

Supplement: Supplementary file 4 — Effects of the anti-CTLA-4 immune checkpoint inhibitor antibody ipilimumab against MC1 PDXs implanted in hNSG mice. Once tumors reached ~ 150 mm3, animals were treated weekly with 10 mg/kg intravenous injections for up to 3 weeks; tumor volumes were evaluated twice weekly. The values represent the mean ± SEM (n = 8). (PPTX 50 kb) [file 13058_2018_1037_MOESM4_ESM.pptx]

## Slide 1
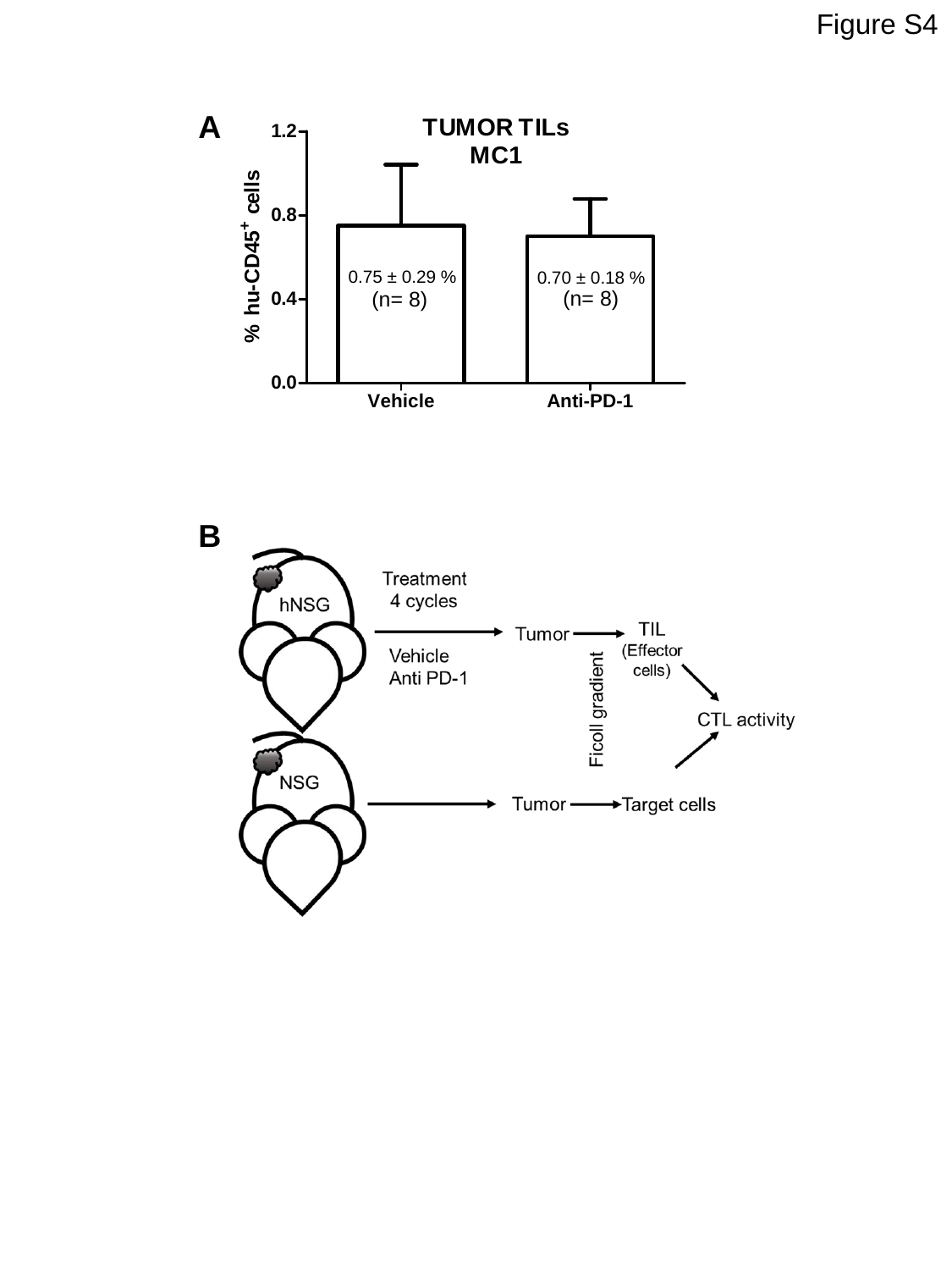

Figure S4
0.75 ± 0.29 %
0.70 ± 0.18 %
A
(n= 8)
(n= 8)
B

Supplement: Supplementary file 5 — a Evaluation of the percentages of human CD45+ TILs present in MC1 PDX tumors engrafted in hNSG mice and collected from animals treated with either vehicle control or anti-PD-1 antibody. The values represent the mean ± SEM (n = 8). b Schematic representation of the method used to determine the cytotoxic activity of TILs by measuring the levels of the lactate dehydrogenase (LDH), a stable cytosolic enzyme that is released upon TIL-induced tumor cell lysis. TILs were isolated from MC1 PDX tumors engrafted in hNSG mice and treated with either vehicle or anti-PD1 antibody that were cocultured with disaggregated MC1 tumor cells obtained from the corresponding PDX grown in nonhumanized NSG mice. (PPTX 131 kb) [file 13058_2018_1037_MOESM5_ESM.pptx]
