# Supplementary material for: Evaluation of anti-PD-1-based therapy against triple-negative breast cancer patient-derived xenograft tumors engrafted in humanized mouse models
Source: Breast Cancer Res. 2018 Sep 5;20:108. doi: 10.1186/s13058-018-1037-4 (PMC6125882; doi:10.1186/s13058-018-1037-4)
Supplement: Supplementary file 2 — Table S1 Analysis of HLA type in PDX BCM-2147/-4913 and CD34+ HSCs. HLA typing was performed by using PCR-SSO DNA-based procedures. The serological phenotype is an interpretation based on molecular typing data. ND Not determined. Table S2 Gene expression analysis (RNA-Seq) comparing MC1, BCM-2147, and BCM-4913 PDXs growing in nonhumanized vs. humanized NSG mice. Differentially expressed genes (DEGs) were selected by edge R-based p value and fold change (FC). Supplemental Methods. (DOCX 22 kb) [file 13058_2018_1037_MOESM2_ESM.docx]

**Table S1**. Analysis of HLA type in PDXs BCM-2147/-4913 and CD34^+^ HSCs. HLA typing was performed by PCR-SSO DNA based procedures. The serological phenotype is an interpretation based on molecular typing data. ND: not determined

|  | **BCM-2147** | **BCM-4913** | **HSC CD34^+^** |
| --- | --- | --- | --- |
| CLASS I | A*29,*29; | A*30,*30; | ND |
|  | B*58,*81; | B*08,*08; | ND |
|  | C*06,*18 | C*03,*03 | ND |
| CLASS II | DRB1*08,*12; | DRB1*07,*07; | ND |
|  | DQA1*01,*04; | DQA1*02,*02; | ND |
|  | DQB1*04,*05; | DQB1*02,*02; | ND |
|  | DRB3*02; | DRB4*01,*01; | ND |
|  | DPB1*18,*18; | DPB1*01,*01; | ND |
|  | DPA1*01,*01 | DPA1*02,*02 | ND |

**Table S2**. Gene expression analysis (RNA-Seq) comparing MC1, BCM-2147 and BCM-4913 PDXs growing in non-humanized vs. humanized NSG mice. Differentially Expressed Genes (DEGs) were selected by edge R based *p* val. and Fold Change (FC).

| **Cut off** | **FC > 1** | **FC > 1.5** | **FC > 2** | **FC > 3** | **FC > 4** |
| --- | --- | --- | --- | --- | --- |
| Adj. *p* val < 0.1 | 8 | 8 | 8 | 8 | 7 |
| Adj. *p* val < 0.05 | 4 | 4 | 4 | 4 | 4 |
| Adj. *p* val < 0.01 | 3 | 3 | 3 | 3 | 3 |
| Adj. *p* val < 0.001 | 2 | 2 | 2 | 2 | 2 |

**Supplemental Methods**

**RNA-Sequence analysis.**

Total RNA was extracted and purified from PDX tumor tissue collected from samples growing in either non-humanized or humanized NSG mice. Approximately 200-250 ng were used for stranded mRNA-Seq library preparation by following the KAPA Stranded RNA-Seq Kit with RiboErase (HMR; Wilmington, MA) sample preparation guide. The first step in the workflow involved the depletion of rRNA by hybridization of complementary DNA oligonucleotides, followed by treatment with RNase H and DNase to remove rRNA duplexed to DNA and original DNA oligonucleotides, respectively. Following rRNA removal, the RNA was fragmented into small pieces using divalent cautions under elevated temperature and magnesium. The cleaved RNA fragments were copied into first strand cDNA using reverse transcriptase and random primers. This was followed by second strand cDNA synthesis using DNA Polymerase I and RNase H. Strand specificity was achieved by replacing dTTP with dUTP in the Second Strand Marking Mix (SMM). The incorporation of dUTP in second strand synthesis effectively quenches the second strand during amplification, since the polymerase used in the assay will not incorporate past this nucleotide. These cDNA fragments then went through an end repair process, the addition of a single ‘A’ base, and then ligation of the adapters. The products were then purified and enriched with PCR to create the final RNA-Seq library. RNA-Seq libraries were subjected to quantification process, pooled for cBot amplification and subsequent sequenced with 50 bp single end sequencing run with Illumina HiSeq 3000 platform. After the sequencing run, demultiplexing with CASAVA will be employed to generate the fastq file for each sample.

All sequencing reads were aligned with their reference genome (UCSC human genome build hg19) using TopHat2 default settings^1,2^ and the BAM files obtained after alignment were processed using HTSeq-count^3^ to obtain the counts per gene in all samples.

**HLA typing**

Low resolution HLA typing was performed by the transplant immunology laboratory at Houston Methodist Hospital.  SSO typing was performed using Lab Type SSO and Lab Type SSO HD reagents from One Lambda (Canoga Park, CA). Testing was performed following the manufacturer’s protocol.  Data were acquired on a Luminex 3D instrument (Luminex, Austin, TX).  HLA typing assignments and bead analysis utilized Fusion 3.2 software (One Lambda, Thermo Fisher Scientific, Waltham, MA).

**Supplemental References**

1. Trapnell, C.*, et al.* Differential analysis of gene regulation at transcript resolution with RNA-seq. *Nat Biotechnol* **31**, 46-53 (2013).

2. Trapnell, C.*, et al.* Differential gene and transcript expression analysis of RNA-seq experiments with TopHat and Cufflinks. *Nat Protoc* **7**, 562-578 (2012).

3. Anders, S. & Huber, W. Differential expression analysis for sequence count data. *Genome biology* **11**, R106 (2010).
